# The distributions of JK blood group system among healthy blood donors in western region of Saudi Arabia: A cross-sectional study

**DOI:** 10.1097/MD.0000000000042677

**Published:** 2025-05-30

**Authors:** Amr J. Halawani, Alaa M. Alkinani, Nedaa A. Alabdali, Ahmed A. Alharbi, Mahmwod S. Qutb Aldeen, Yasser A. Albalwi, Anas K. Alghaith, Mohammed A. Bamarouf, Mada M. Alqarni, Maryam A. Munshi, Shuroog S. Alessa, Duaa I. Bukhari, Raghad M. Alaslani, Noora S. Bakhashwain, Abrar H. AlHarbi, Waleed AlZubaidi, Sahal A. Jamalallail

**Affiliations:** a Department of Clinical Laboratory Sciences, Faculty of Applied Medical Sciences, Umm Al-Qura University, Makkah, Saudi Arabia; b King Abdulaziz Medical City, Ministry of National Guard Health Affairs, Jeddah, Saudi Arabia.

**Keywords:** alloimmunization, blood transfusion, JK blood group, Saudi Arabia

## Abstract

The Kidd (JK) blood group system has 3 antigens: Jka, Jkb, and Jk3. The forming antibodies directed against these antigens can lead to hemolytic transfusion reactions due to incompatible blood transfusions. This study aimed to determine the incidence of Jka and Jkb antigens and their phenotypes among blood donors at King Abdulaziz Medical City-Western Region, Saudi Arabia. In this cross-sectional retrospective study, a total of 14,987 blood donors were attended at the blood bank of King Abdulaziz Medical City-Western Region between January 2020 and May 2024. Serological investigation was conducted using a solid-phase technique to identify the Jka and Jkb antigens as well as the phenotypes. Most blood donors were Saudis (n = 12,844, 85.70%), whereas 2143 (14.30%) were non-Saudis. The distributions of Jka and Jkb antigens in Saudis were 87.87% and 53.94%, respectively. The frequencies in the non-Saudi population were 84.10% and 64.39%, respectively. This difference was statistically significant (*P* < .01). The proportions of the JK phenotypes showed a highly statistically significant difference between Saudis and non-Saudis (*P* < .01). Among Saudis, the JK phenotypes were 46.04% Jk(a+b−), 41.48% Jk(a+b+), 12.09% Jk(a−b+). and 0.03% Jk(a−b−). In contrast, the JK phenotypes in non-Saudis were 35.60% Jk(a+b−), 48.53% Jk(a+b+), and 15.87% Jk(a−b+), with no incidence of Jk(a−b−) phenotype. This study reported the frequencies of JK blood group among Saudi and non-Saudi blood donors. Based on these findings, incorporation of JK antigen screening into transfusion protocols is recommended to enhance the safety and efficacy of blood transfusions.

## 1. Introduction

The Kidd (JK) blood group system was revealed in 1951 after a mother “Mrs. Kidd” formed an antibody against Jka antigen, the first identified antigen in the system. Her baby suffered from hemolytic disease of the fetus and newborn (HDFN), leading to the discovery of this blood group.^[1]^ The JK system consists of 3 antigens: 2 antithetical antigens (Jka and Jkb), and a third antigen, Jk3. It has been classified as a blood group system 009 by the International Society of Blood Transfusion.^[2]^

The resultant JK antigens on the red cell surface are encoded by *human urea transporter 11 gene*, located on chromosome 18. This gene comprises 11 exons and spans approximately 30 kb of DNA. It produces 3 main alleles, which are JK*A, JK*B, and the nonfunctional allele *JK.*^[3]^ A single nucleotide variant (c.838C > G) in exon 9 of the *human urea transporter 11* gene distinguishes the JK*A allele from JK*B allele, leading to an amino acid substitution (p.Asn280Asp) that alters the protein structure.^[4]^

The JK glycoprotein is composed of 389 amino acids and spans the red cell membrane 10 times, forming 5 extracellular loops that include 2 glycosylation sites.^[5]^ The fourth extracellular loop contains Jka and Jkb antigens. This glycoprotein may function as a urea transporter, playing a crucial role in preserving the structural integrity of red cells.^[6]^

Four JK phenotypes can be observed: Jk(a+b−), Jk(a+b+), Jk(a−b+), and Jk(a−b−). The JK_null_ phenotype, Jk(a−b−), was first observed in a Filipino woman who experienced jaundice following blood transfusion. This phenotype is rare, but is most prevalent in Finnish and Polynesian ethnicities.^[7]^ The JKnull phenotype arises from deletions or SNV, leading to a nonfunctional protein on the red cell surface.^[8]^

JK antibodies can cause immediate and delayed hemolytic transfusion reactions (HTRs). Anti-Jka antibodies are typically associated with mild-to-moderate HTRs, although severe reactions may occur, resulting in significant morbidity.^[9]^ The anti-Jkb and anti-Jk3 antibodies are less common. However, these can lead to severe immediate or delayed HTRs.^[10]^ Furthermore, several cases of HDFN have been reported. Although JK HDFN is often mild, it can be severe or even fatal.^[11–13]^

In addition to the blood transfusion challenges posed by the JK blood group, it has a potential role as a minor histocompatibility antigen. Anti-Jka and anti-Jkb antibodies have been linked to acute renal transplant rejection.^[14,15]^

Given the importance of the clinical significance of JK antibodies and their impact on transfusion practices, the aims of the present study was to screen the incidence of the JK antigens as well as the 4 phenotypes in Saudi and non-Saudi blood donors in the King Abdulaziz Medical City-Western Region (KAMC-WR).

## 2. Materials and Methods

### 2.1. Participants and study design

This cross-sectional retrospective study included 14,987 healthy blood donors representing eligibility of blood donation between January 2020 and May 2024 at the blood bank center of KAMC-WR, Jeddah City, Saudi Arabia. The current study was carried out in compliance with the Declaration of Helsinki.

Repeat donors were excluded using the donor identification number to ensure that each blood donor was only represented once to determine the actual prevalence among individual donors. Any successful blood donations that were free from infectious diseases were included in this study.

### 2.2. Testing and result interpretation

Serological analysis, based on a solid-phase technique, was performed using Gamma-clone anti-Jka and anti-Jkb monoclonal antibodies to detect Jka and Jkb antigens, according to the manufacturer’s instructions (Immucor Medizinische Diagnostik GmbH, Dreieich, Germany). The agglutination assay yielded a positive result, indicating the presence of either Jka or Jkb antigens on the corresponding red cells.

### 2.3. Interpretation of results

Positive test results were indicated by agglutination of the tested red cells with monoclonal antibodies, confirming the presence of the corresponding antigen. In contrast, the absence of agglutination signifies negative results, indicating that the antigen was not expressed on the red cell surface.

### 2.4. Statistical analysis

A total of 664 samples were estimated using the Raosoft Sample Size Calculator with a 99% confidence level and 5% margin of error. The frequencies of JK blood groups for Saudi and non-Saudi donors were determined and standardized as percentages. The chi-squared test was used to detect statistically significant outcomes. *P*-values of< .01 designated to highly significant differences.

### 2.5. Ethical aspects

This study was approved by the Institutional Review Board of the King Abdullah International Medical Research Center (No. 0000086124), Ministry of National Guard Health Affairs, Kingdom of Saudi Arabia. Informed consent from the blood donors was waived because of the retrospective nature of the study.

## 3. Results

In this study, 14,987 blood donors were examined for JK antigens and phenotypes. The majority of blood donors were Saudis (n = 12,844, 85.70%), while the remainder were non-Saudis (n = 2143, 14.30%), as shown in Table 1.

**Table 1:** Sociodemographic data of the blood donors in the present study (n = 14,987).

Nationality	n	%
Saudi	12,844	85.70
Non-Saudi	2143	14.30
Total	14,987	100

Table 2 lists the distributions of the JK antigens in both populations, Saudis and non-Saudis. The rates of the Jka and Jkb antigens in Saudis were 87.87% and 53.94%, respectively. On the other hand, the distributions for these 2 antigens in non-Saudis were expressed at 84.10% and 64.39%, respectively.

**Table 2:** Distributions of JK antigens in Saudi Arabians and non-Saudi Arabians.

	Saudi Arabians (n = 12,844)		Non-Saudi Arabians (n = 2143)		Chi-square	*P*-value
Antigen	Observation	Frequency (%)	Observation	Frequency (%)	32.11†	.001*
Jka	11,287	87.87	1803	84.10		
Jkb	6927	53.94	1380	64.39		

JK = Kidd.

* Highly significant (*P* < .01).

† Chi-square test statistic.

Four JK phenotypes were determined for Saudis and non-Saudis (Table 3). The JK phenotypes in Saudi Arabia are as follows: 46.06% Jk(a+b−), 41.84% Jk(a+b+), 12.09% Jk(a−b+), and 0.03% Jk(a−b−). However, the phenotypes in non-Saudis were 35.60% Jk(a+b−), 48.53% Jk(a+b+), 15.87% Jk(a−b+), and there was no incidence for the JK_null_ phenotype. Variations can be observed, especially in Saudi Arabian populations, among the different provinces of Saudi Arabia, as indicated in Table 4.

**Table 3:** Comparison of distribution of JK phenotypes between Saudi Arabian population versus non-Saudi Arabians.

	Saudi Arabians (n = 12,844)		Non-Saudi Arabians (n = 2143)		Chi-square	*P*-value
Phenotype	Observation	Frequency (%)	Observation	Frequency (%)	85.46†	.001*
Jk(a+b−)	5913	46.04	763	35.60		
Jk(a+b+)	5374	41.84	1040	48.53		
Jk(a−b+)	1553	12.09	340	15.87		
Jk(a−b−)	4	0.03	0	0.00		
	12,844	100	2143	100		

JK = Kidd

* Highly significant (*P* < .01).

† Chi-square test statistic.

**Table 4:** JK phenotype distributions in the Saudi population in the present study in comparison with different provinces in Saudi Arabia.

Phenotype	Current (%) n = 12,844	Jazan^[16]^ (%) n = 143	Eastern^[17]^ (%) n = 100	Al-Ahsa^[18]^ (%) n = 1549
Jk(a+b−)	46.04	34.96	40	27.6
Jk(a+b+)	41.84	52.45	46	31
Jk(a−b+)	12.09	12.59	14	41.3
Jk(a−b−)	0.03	0	0	0.1
†*P*-values		Current/Jazan *P* = .27	Current/Eastern *P* = .68	Current/Al-Ahsa *P* = .001*

JK = Kidd

* Highly significant (*P* < .01).

† Chi-square test statistic.

## 4. Discussion

It is crucial to understand the distribution of different blood groups to preclude HTRs, especially in areas endemic to poly-transfused patients, such as patients with thalassemia and sickle cell disease.^[19–21]^ Because of repeated exposure to blood transfusions, these patients tend to form alloantibodies directed against the red cell antigens they lack.^[22]^ As a result, many studies have been conducted to investigate the prevalence of various blood group antigens to avoid the risk of red cell alloimmunization.^[16,23–31]^

This study identified the prevalence of the Jka and Jkb antigens of the JK blood group system in Saudi and non-Saudi populations enrolled in the KAMC-WR for blood donation. The distributions of Jka and Jkb in Saudis were 87.87% and 53.94%, respectively. A statistically significant difference was observed in JK antigens between the Saudi and non-Saudi populations (*P* < .01) as demonstrated in Table 2.

Regarding the JK phenotypes, the most prevalent among Saudis was Jk(a+b−) at 46.06%. On the other hand, the most common among non-Saudis was Jk(a+b+), at 48.53%. There was a statistically significant difference in the JK phenotypes between Saudi and non-Saudi individuals (*P* < .01; Table 3).

The prevalence of the Jk(a+b−) phenotype in our study was observed in 46.06% of Saudi donors, which was less than that found in Saudis living in Eastern Province (40%),^[17]^ yet higher than that in Saudis living in Jazan Province (34.96%; Table 4).^[16]^ The most common JK phenotype among Saudis in Jazan and Eastern Provinces was homozygous Jk(a+b+), at 52.45% and 46%, respectively.^[16,17]^

Remarkably, the JK phenotypes of the present study were observed highly statistical difference in the Saudi population compared to Al-Ahsa Province of Saudi Arabia (*P* < .01).^[18]^ The most prevalent JK phenotype in A-Ahsa region was Jk(a−b+), accounting for 41.3%. Interestingly, the rarest JK phenotype “Jk(a−b−)” was observed in Saudis in our study (0.03%) and it was significantly lower than the reported finding in Al-Ahsa Province (0.1%) as presented in Table 4.^[18]^ These findings emphasize regional variations in antigen expression across Saudi Arabia, demonstrating that country’s diverse ethnic composition.

It is highly recommended that transfusion practices in Saudi Arabia include screening for JK antigens in both blood donors and recipients, regardless of nationality. This is particularly essential for transfusion-dependent patients to preclude the risk of the red cell alloimmunization.^[32,33]^

A limitation of this lies in the lack of testing for blood group antigens beyond Jka and Jkb. Nevertheless, the study’s findings remain robust due to the large sample size.

## 5. Conclusion

In summary, the distributions of JK blood group antigens as well as phenotypes were identified in both Saudi and non-Saudi individuals in the KAMC-WR. The most prevalent phenotype among Saudis was Jk(a+b−), whereas the Jk(a+b+) phenotype was the most common among non-Saudis. These variations should be considered in transfusion practice by including JK antigen testing in the screening panel to help prevent red cell alloimmunization. Furthermore, it is highly recommended that such research be conducted regarding the prevalence of various blood groups in the different provinces of Saudi Arabia.

## Acknowledgments

The authors extend their appreciation to Umm Al-Qura University, Saudi Arabia for funding this research work through grant number: 25UQU4420107GSSR02.

## Author contributions

**Conceptualization:** Duaa I. Bukhari, Noora S. Bakhashwain, Abrar H. AlHarbi, Sahal A Jamalallail.

**Data curation:** Alaa M. Alkinani, Nedaa A. Alabdali, Ahmed A. Alharbi, Mahmwod S. Qutb Aldeen, Yasser A. Albalwi, Anas K. Alghaith, Mohammed A. Bamarouf, Mada M. Alqarni, Maryam A. Munshi, Shuroog S. Alessa, Raghad M. Alaslani, Waleed AlZubaidi, Sahal A Jamalallail.

**Formal analysis:** Sahal A Jamalallail.

**Funding acquisition:** Amr J. Halawani.

**Supervision:** Amr J. Halawani, Sahal A Jamalallail.

**Validation:** Sahal A Jamalallail.

**Writing – original draft:** Amr J. Halawani.

**Writing – review & editing:** Amr J. Halawani, Alaa M. Alkinani, Nedaa A. Alabdali, Ahmed A. Alharbi, Mahmwod S. Qutb Aldeen, Yasser A. Albalwi, Anas K. Alghaith, Mohammed A. Bamarouf, Mada M. Alqarni, Maryam A. Munshi, Shuroog S. Alessa, Duaa I. Bukhari, Raghad M. Alaslani, Noora S. Bakhashwain, Abrar H. AlHarbi, Waleed AlZubaidi, Sahal A Jamalallail.
